# Prescription Department

**Published:** 1897-08

**Authors:** 


MIXTURE FOR THE TREATMENT OF WHOOPING-COUGH.

℞. Tinct. of belladonna...10 grms.
Phenacetin............5 grms.
Rye whiskey...........15 grms.
Fluid extract of chestnut leaves.............. 60 grms.

M. Sig: Mix. Ten drops (for children of one year) to a teaspoonful (for children ten years of age) every two to six hours. DR R. A. LANCASTER.

FOR CHRONIC INTERSTITIAL NEPHRITIS.

℞. Hydrarg cor. chlor........gr. i.

Tr. ferri chlor.............. ʒij.

Sp. eth. nitros............. ℥ij.

Aquæ...................... ℥iss.

Elix. simp. ad... .....℥iv.

M. Sig: Teaspoonful in water after each meal.—*N. E. Medical Monthly*.

